# Anti-obesity and anti-inflammatory effects of synthetic acetic acid vinegar and Nipa vinegar on high-fat-diet-induced obese mice

**DOI:** 10.1038/s41598-017-06235-7

**Published:** 2017-07-27

**Authors:** Boon Kee Beh, Nurul Elyani Mohamad, Swee Keong Yeap, Huynh Ky, Sook Yee Boo, Joelle Yi Heng Chua, Sheau Wei Tan, Wan Yong Ho, Shaiful Adzni Sharifuddin, Kamariah Long, Noorjahan Banu Alitheen

**Affiliations:** 1 Biotechnology Research Centre, Malaysian Agricultural Research and Development Institute (MARDI), Serdang, Selangor 43400 Malaysia; 2 Institute of Bioscience, Universiti Putra Malaysia, Serdang, Selangor Malaysia; 3 Department of Cell and Molecular Biology, Faculty of Biotechnology and Biomolecular Science, Universiti Putra Malaysia, Serdang, Selangor 43400 Malaysia; 4 China-ASEAN College of Marine Sciences, Xiamen University Malaysia, Jalan Sunsuria, Bandar Sunsuria, 43900 Sepang, Selangor Malaysia; 5 Department of Agriculture Genetics and Breeding, College of Agriculture and Applied Biology, Cantho University, Can Tho, 84071 Vietnam; 6 BioEasy Sdn Bhd, Setia Alam, Shah Alam, Selangor Malaysia; 7 School of Biomedical Sciences, The University of Nottingham Malaysia Campus, Jalan Broga, 43500 Semenyih, Selangor Malaysia

## Abstract

Recently, food-based bioactive ingredients, such as vinegar, have been proposed as a potential solution to overcome the global obesity epidemic. Although acetic acid has been identified as the main component in vinegar that contributes to its anti-obesity effect, reports have shown that vinegar produced from different starting materials possess different degrees of bioactivity. This study was performed to compare the anti-obesity and anti-inflammatory effects of synthetic acetic acid vinegar and Nipa vinegar in mice fed a high-fat diet. In this work, mice were fed a high-fat diet for 33 weeks. At the start of week 24, obese mice were orally fed synthetic acetic acid vinegar or Nipa vinegar (0.08 and 2 ml/kg BW) until the end of week 33. Mice fed a standard pellet diet served as a control. Although both synthetic acetic acid vinegar and Nipa vinegar effectively reduced food intake and body weight, a high dose of Nipa vinegar more effectively reduced lipid deposition, improved the serum lipid profile, increased adipokine expression and suppressed inflammation in the obese mice. Thus, a high dose of Nipa vinegar may potentially alleviate obesity by altering the lipid metabolism, inflammation and gut microbe composition in high-fat-diet-induced obese mice.

## Introduction

Obesity caused by long-term energy imbalance is an epidemic disease in developed and developing countries^1^. This chronic disease is one of the major criteria for metabolic syndrome and is also associated with high blood pressure, hyperlipidaemia, insulin resistance and proinflammatory status^2^. To overcome these problems, better prevention and treatment options with minimum or zero side effects compared to currently available medicines are needed. In addition to the control of daily calorie intake and a more active lifestyle, consuming diets or food with bioactive nutrients has been proposed as an effective solution to combat and even prevent obesity^3^.

Microbial fermentation is a food-processing strategy that is commonly used to process foods. The resulting fermented food, which is known to be healthy and beneficial, has been shown to possess enhanced nutritional value and health benefits because of the production of unique organic compounds and secondary metabolites by microbiological action^4^. For example, fermented food has been demonstrated to exhibit anti-obesity effects through altering gut microbiota composition and the expression of genes related to metabolic syndrome^5^. Among these fermented foods, vinegar, an acidic food seasoning, has recently received substantial attention because it was shown to exhibit multiple bioactivities, including anti-hypercholesterolaemia, anti-hyperglycaemia, anti-hypertension, anti-microbial, anti-thrombotic and even anti-cancer effects^6^. Acetic acid, which is the major ingredient in vinegar, has been reported to be a potential agent for preventing metabolic syndrome by reducing obesity in rats^7^ and even obese human subjects^8^. In addition, vinegar has also been shown to improve serum glucose levels and insulin resistance in patients with type 1^9^ and type 2 diabetes^10–13^. However, Nishidai *et al*.^14^ reported that the health benefits of different types of vinegar can vary because of the different levels of antioxidants contributed by the carbohydrate source and microbial strain used for vinegar fermentation.

Nipa vinegar is commonly used as a food additive and in folk medicine to treat diabetes and high blood pressure^15^. However, the health benefits of Nipa vinegar, especially its anti-obesity effects, have not been evaluated previously. Additionally, the alteration of gut microbiota and regulation of the expression of genes related to metabolic syndrome by synthetic acetic acid vinegar and Nipa vinegar remain unknown. Therefore, this study was conducted to compare the anti-obesity and anti-inflammatory effects of synthetic acetic acid vinegar and Nipa vinegar in high-fat diet (HFD)-induced obese mice.

## Results

### A high dose of vinegar reduces the body weight (BW) and fat pad/BW ratio of obese mice

**Figure 1:**
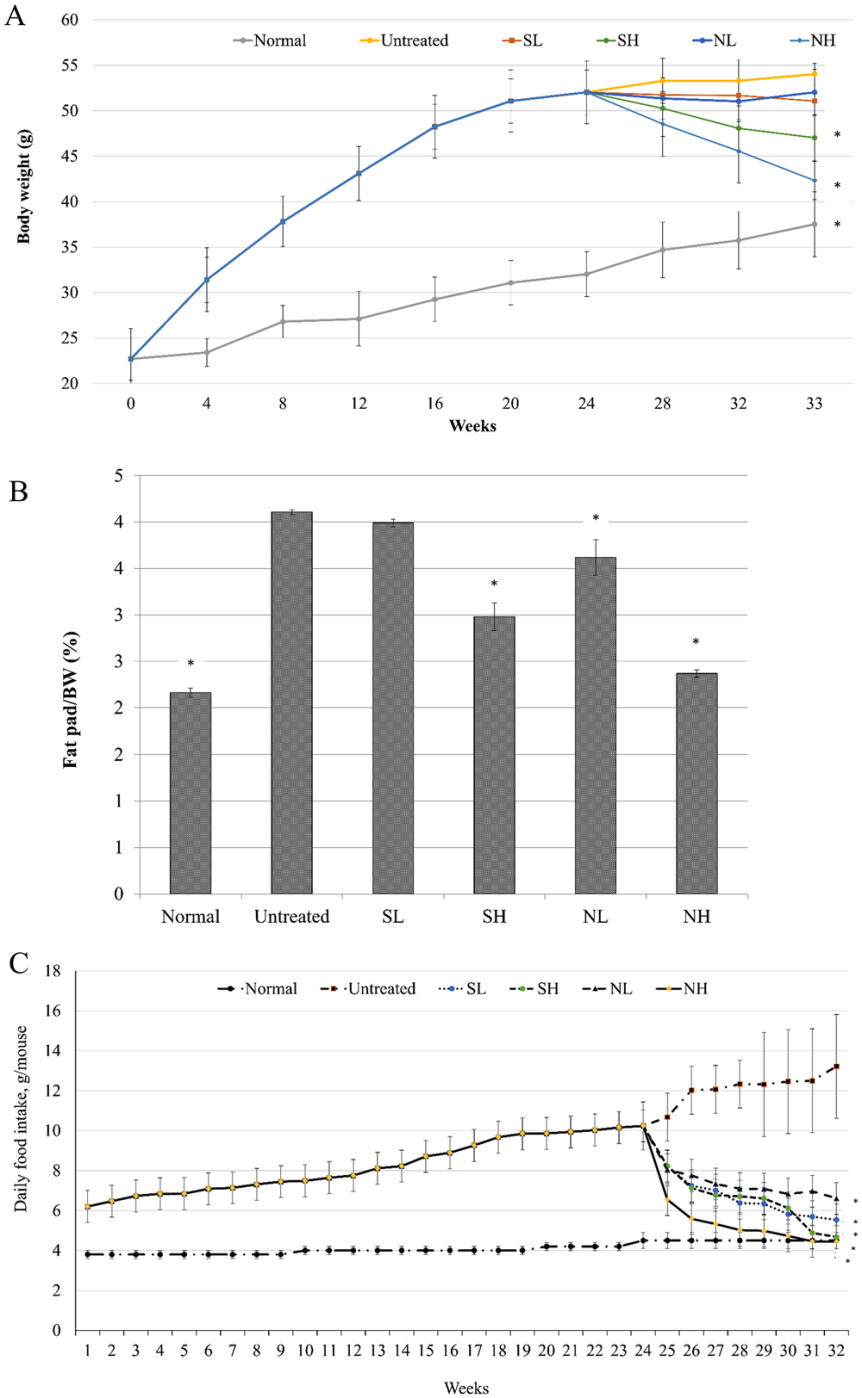
(**A**) BW measurements (week 0-week 33) of normal healthy mice, untreated mice,and 0.08-ml/kg BW synthetic acetic acid vinegar (SL)-, 2-ml/kg BW synthetic acetic acid vinegar (SH)-, 0.08-ml/kg BW Nipa vinegar (NL)- and 2-ml/kg BW Nipa vinegar (NH)-treated obese mice. Vinegar treatment was started at week 24 and ended at the end of week 33 (10 weeks). (**B**) Fat pad/BW ratios of normal healthy, untreated, and 0.08-ml/kg BW (SL)-, 2-ml/kg BW (SH), 0.08-ml/kg BW (NL) and 2-ml/kg BW (NH)-treated obese mice at week 33. (**C**) Food intake measurements of normal healthy, untreated, and 0.08-ml/kg BW (SL), 2-ml/kg BW (SH), 0.08-ml/kg BW (NL) and 2-ml/kg BW (NH)-treated obese mice. The data are presented as averages of biological replicates (n = 6) from the same treatment group. Values marked with * differed significantly compared to untreated groups (p < 0.05).

Nipa vinegar was standardized to 4% acetic acid, contains gallic acid (143 µg/ml) and protocatechuic acid (61 µg/ml), which contribute to its enhancement of liver superoxide dismutase (SOD), glutathione (GSH) and ferric reducing antioxidant power (FRAP) and suppression of nuclear factor kappa-light-chain-enhancer of activated B cells (NF-κB)^16^. C57BL/6 mice fed an HFD had a recorded weight of >50 g/mouse (Fig. 1A) and a fat pad/BW ratio of 4% (Fig. 1B). Mice fed a normal diet had a BW of ~30 g/mouse (Fig. 1A), a fat pad/BW ratio of ~2% (Fig. 1B) and daily food intake of ~4.8 g/mouse (Fig. 1C) after 33 weeks of incubation, which were significantly lower than the untreated obese mice. After 10 weeks of treatment, the obese mice treated with 2-ml/kg BW synthetic acetic acid vinegar (SH) and 2-ml/kg BW Nipa vinegar (NH) exhibited statistically significant lower body weight and fat pad/BW ratio (p < 0.05) relative to untreated obese group (Fig. 1A and B). On the other hand, 0.08-ml/kg BW synthetic acetic acid vinegar (SL) treatment did not significantly reduced the body weight and fat pad/BW ratio relative to untreated obese group (Fig. 1A and B). In addition, 0.08-ml/kg BW Nipa vinegar (NL)-treatment only significantly (p < 0.05) reduced the fat pad/BW ratio but no significant effect on body weight changes relative to untreated obese group. This result may be attributable to reduced food intake (Fig. 1C) because the treatments resulted in significant suppression of food intake by either synthetic vinegar or nipa vinegar (p < 0.05) relative to the untreated obese group. However, suppression of the food intake only partially contributed to reduce of body weight and fat pad/BW ratio, as SL and NL treatments, which also significantly (p < 0.05) suppress the food intake did not observe with significant different of body weight changes relative to the obese untreated group.

### A high dose of vinegar improved the serum lipid profiles of obese mice

**Figure 2:**
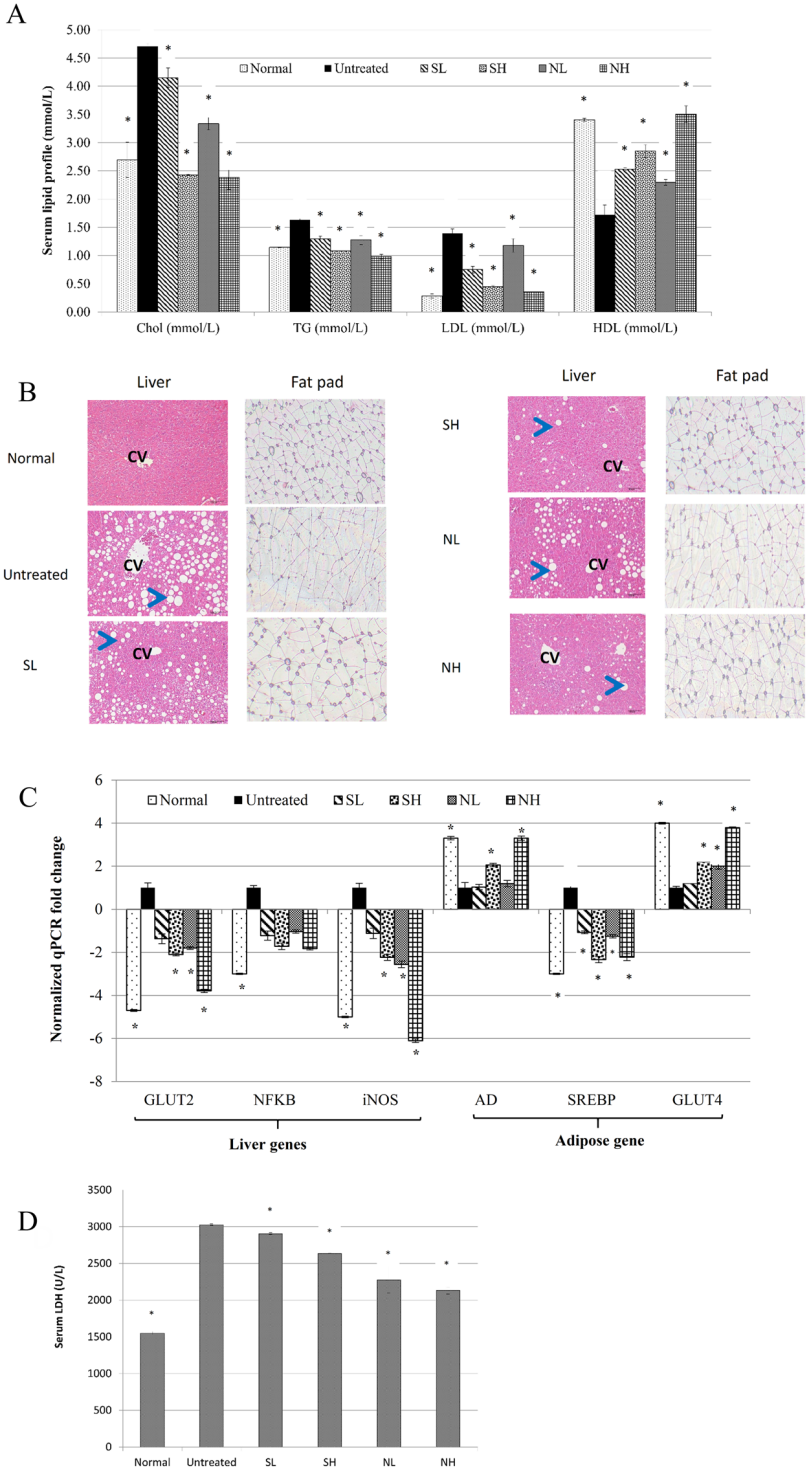
(**A**) Serum lipid profile (total cholesterol, total TG, LDL and HDL). (**B**) Representative histopathological analyses of the liver and fat pad (CV represents the central vain; the arrow indicates a lipid droplet; magnification: 100x). (**C**) qPCR analyses of liver inflammation and obesity-related gene expression. (**D**) Serum LDH levels of normal healthy, untreated, and 0.08-ml/kg BW synthetic acetic acid vinegar (SL), 2-ml/kg BW synthetic acetic acid vinegar (SH), 0.08-ml/kg BW Nipa vinegar (NL) and 2-ml/kg BW Nipa vinegar NH-treated obese mice at week 33. The data are presented as averages of biological replicates (n = 6) from the same treatment group. Significant values were calculated relative to the untreated group (*p < 0.05). The data are presented as averages of biological replicates (n = 6) from the same treatment group. Values marked with * differed significantly compared to untreated groups (p < 0.05).

C57BL/6 mice fed an HFD were recorded with significantly higher serum lipid profile (Fig. 2A), leptin, glucose and insulin levels (Table 1) than the normal mice. Obese mice treated with both concentration of synthetic acetic acid vinegar and nipa vinegar exhibited statistically significant (p < 0.05) lower serum total cholesterol, triglyceride (TG), low-density lipoprotein (LDL) and higher high-density lipoprotein (HDL) (p < 0.05) relative to the untreated obese group. In addition, NH treatment significantly (p < 0.05) reduced serum leptin, glucose and insulin level while SH treatment only significantly (p < 0.05) reduced the serum leptin level relative to untreated obese group. No significant difference was observed for the serum total cholesterol, TG, LDL and HDL of the SL or NL treated groups relative to the untreated group (Table 1). This result was strongly supported by the liver and fat pad histopathological analyses in which the obese mice treated with SH and NH exhibited smaller adipocyte cell sizes and fewer lipid droplets in the liver compared to the untreated obese mice (Fig. 2B). These effects were observed less frequently with SL- and NL-treated obese mice.

**Table 1:** Serum leptin, glucose insulin, and liver/fat pad NO levels of normal healthy, untreated, 0.08-ml/kg BW synthetic acetic acid vinegar (SL)-treated, 2-ml/kg BW synthetic acetic acid vinegar (SH)-treated, 0.08-ml/kg BW Nipa vinegar (NL)-treated and 2-ml/kg BW Nipa vinegar NH-treated obese mice.

	Leptin (ng/mL)	Glucose (mmol/L)	Insulin (ng/mL)	NO (µM NO/mg protein)
	Serum	Serum	Serum	Liver
Normal	8.23 ± 1.33^*^	4.10 ± 0.31^*^	0.91 ± 0.26^*^	4.64 ± 0.50^*^
Untreated	33.82 ± 2.96	7.10 ± 0.56	2.10 ± 0.46	6.27 ± 0.61
SL	31.32 ± 2.76	6.62 ± 0.33	1.84 ± 0.36	4.15 ± 0.48^*^
SH	19.97 ± 2.87^*^	5.97 ± 0.45	1.68 ± 0.37	2.88 ± 0.46^*^
NL	29.71 ± 3.27	6.65 ± 0.47	1.89 ± 0.31	2.60 ± 0.15^*^
NH	12.45 ± 4.52^*^	4.88 ± 0.66^*^	1.13 ± 0.24^*^	2.46 ± 0.14^*^

The data presented are represented as the mean ± SD of biological replicates of mice from the same treatment group. Values marked with * differed significantly compared to untreated groups (p < 0.05).

### Nipa vinegar reduced inflammation in obese mice

The anti-inflammatory effect of Nipa vinegar in obese mice was evaluated by quantifying the mRNA expression level of NF-κB and inducible nitric oxide (NO) synthase (iNOS) genes in the liver. Normal control mice were observed with significantly (p < 0.05) lower expression of liver iNOS and NF- κB and liver NO level relative to untreated obese mice. SH, NL and NH treatments significantly (p < 0.05) suppressed the expression of iNOS in the liver relative to the untreated obese group. However, the expression of NF-κB was not significantly down-regulated by SH, NL and NH treatments (<2-fold difference compared to the untreated group). SL did not significantly regulate either iNOS or NF-kB mRNA expression in the liver (<2-fold difference compared to the untreated group) (Fig. 2C). The anti-inflammatory effects of the treatments were further supported by an NO assay in which the NO level in the liver was significantly (p < 0.05) reduced by SH, NL and NH treatments compared to the untreated obese group (Table 1).

The suppression of inflammation, particularly by Nipa vinegar, contributed to better protection against HFD-induced liver damage, as indicated by significant reduction in the serum lactate dehydrogenase (LDH) level by synthetic acetic acid vinegar and nipa vinegar treatments in dosage dependent manner (p < 0.05) relative to obese mice (Fig. 2D).

### High concentrations of both vinegars regulated the expression of hepatic glucose transporter type 2 (GLUT2) and adipose GLUT4, adiponectin (AD) and sterol regulatory element-binding protein 1 (SREBP1) mRNA expression

Normal control mice significantly (p < 0.05) overexpressed AD and GLUT4 (adipose) and suppressed expression of SREBP (adipose) and GLUT2 (liver) relative to untreated obese mice. The relative mRNA expression of GLUT4 and AD was significant up-regulated by both SH and NH treatment (p < 0.05), in the adipose tissue. In contrast, SREBP1 in adipose and GLUT2 in the liver were significantly down-regulated by both SH and NH treatment (p < 0.05). SL did not significantly alter the expression of these genes (<2-fold difference compared to the untreated group) while NL only significantly downregulated expression of liver GLUT2 and upregulated expression of adipose GLUT4 (Fig. 2C).

### High concentrations of both vinegars altered the gut microbiota composition of the obese mice

**Figure 3:**
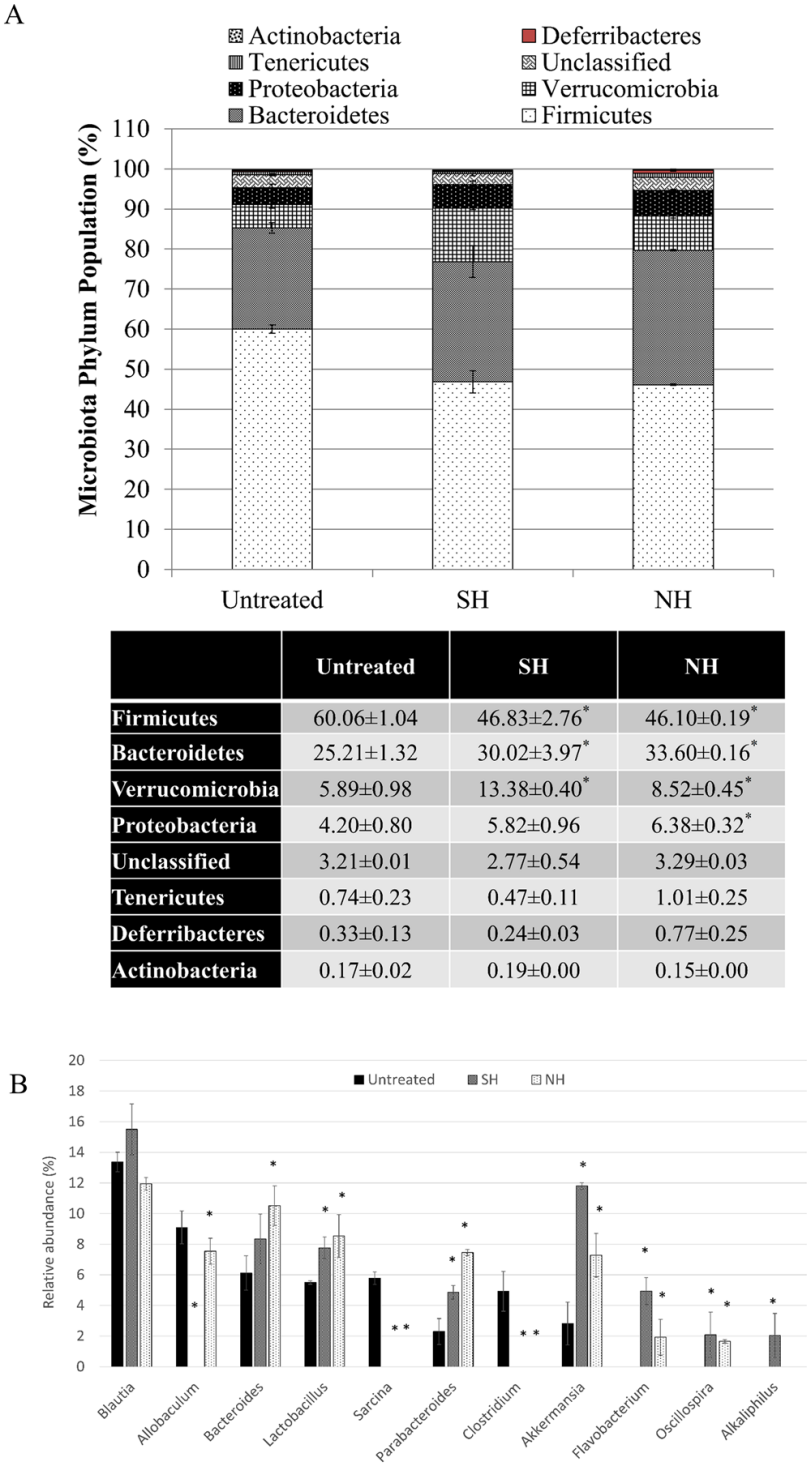
Comparison of the relative abundances of gut microbiota at the (**A**) phylum and (**B**) genus levels of untreated, 2-ml/kg BW synthetic acetic acid vinegar (SH), and 2-ml/kg BW Nipa vinegar (NH)-treated obese mice at week 33 post-experiment using Illumina 16S rRNA metagenomics sequencing. The data are presented as the mean ± SD of biological replicates (n = 3) from the same treatment group. Values marked with * differed significantly compared to untreated groups (p < 0.05).

Firmicutes were the most dominant phylum population in the gut microbiota of untreated obese mice. Both SH and NH resulted in significant (p < 0.05) decreases in the gut Firmicutes/Bacteroidetes ratio. Furthermore, significantly (p < 0.05) increased Verrucomicrobia and Proteobacteriaphylum populations were observed after SH and NH interventions (Fig. 3A). The relative abundances of *Bacteroides, Lactobacillus, Parabacteroides, Akkermansia, Flavobacterium* and *Oscillospira* increased significantly (p < 0.05), whereas those of *Allobaculum, Sarcina, Clostridium* decreased significantly (p < 0.05) after 10 weeks of SH and NH treatments (Fig. 3B).

## Discussion

Obesity, which is primarily attributable to a prolonged imbalance between energy intake and energy expenditure, is a prevalent health issue worldwide and is associated with modernization. Although genetics and environmental factors are major causes of obesity, modern eating habits, especially consuming HFDs, have been identified as the most important reason for the increased risk of obesity^17^. HFD-induced obesity in mice has been reported as a good model for human obesity because it is associated with liver fat accumulation; elevated BWs, lipid profiles, lipid peroxidation, inflammation, insulin and leptin secretion; and decreased body antioxidant levels^18^, similar to the characteristics observed in HFD-induced untreated obese mice in this study. To overcome this disease without side effects, finding an effective treatment based on natural products, especially those developed using food sources, is a major focus^5^. Fermented foods, especially vinegars produced from different crops or fruits, are consumed worldwide to obtain various health benefits, including weight regulation. For example, tomato vinegar has been reported to have anti-obesity effects in HFD-induced mice^19^. Although acetic acid has been proposed to be the main vinegar ingredient responsible for this anti-obesity effect^7^, different vinegars have manifested different bioactivities^14^. Therefore, the anti-obesity effects and ability to regulate the gut microbiota exhibited by synthetic acetic acid vinegar and Nipa vinegar in HFD-induced obese mice were evaluated in this study. A review revealed that the acetic acid in vinegar possesses anti-obesity and anti-glycaemic effects, predominantly by increasing satiety and thereby reducing the total amount of food consumed^20^. This effect is similar to those of treatment with 2-ml/kg BW synthetic acetic acid vinegar (SH) and Nipa vinegar (NH) observed in this study (Fig. 1C). Interfering with lipid mobilization and lipid deposition has been proposed as an effective way to treat obesity^18^. SH and NH were observed to reduce lipid mobilization and deposition, thereby helping to reduce BW. Interestingly, NH exhibited an anti-obesity effect similar to that of tomato vinegar but at a 7-fold lower volume^19^.

Because the lipid metabolism involved in preadipocyte differentiation is associated with the expression of genes controlling lipogenesis and lipolysis, such as leptin, AD and SREBP1 transcriptional factor^21^, the ability of synthetic acetic acid vinegar and Nipa vinegar to regulate these obesity-related genes was evaluated. Leptin is a protein produced primarily by adipocytes, and serum leptin levels correlate with adipocyte fat storage^22^. Hence, the reduction in serum leptin levels caused by both vinegars was attributable to the reduction of the adipocyte size and body fat accumulation. Reducing the leptin level has been linked to satiety^22^, and the reduction in leptin that results from the consumption of both vinegars was determined to be associated with reduced food intake in both SH- and NH-treated obese mice. In parallel with the down-regulation of leptin, the expression of the SREBP1 gene was down-regulated by both SH and NH treatments. SREBP1 is a transcription factor involved in activating lipids and carbohydrate metabolism through glucose uptake^18^. Mediating the accumulation of lipid droplets and cholesterol increased the serum lipid profile^23^. Both vinegars decreased fat accumulation in the liver, serum cholesterol and TG levels by down-regulating the expression of the SREBP1 gene and serum leptin level, which in turn inhibited lipogenesis and overcame the insulin resistance caused by HFD.

Unlike leptin, AD is responsible for regulating glucose and fatty acid oxidation in the body. A low level of AD, which is an antagonist of adipogenesis by adipocytes, was accompanied by the development of insulin resistance and obesity^24^. Moreover, adipose GLUT4 and hepatic GLUT2 are insulin-regulated glucose transporters that help maintain glucose homeostasis. The insulin resistance of obese mice has consistently been determined to be related to the down-regulation of adipose GLUT4 and the overexpression of hepatic GLUT2, suggesting that the dysregulation of glucose homeostasis and increased hepatic glucose output, respectively, play a role in the development of this condition^25^. NH resulted in the up-regulation of adipose tissue AD and GLUT4 expression and down-regulation of GLUT2 in the livers of obese mice. Thus, Nipa vinegar sensitized glucose transport and metabolism in the liver, which is associated with more effective weight management, compared to synthetic acetic acid vinegar.

Prolonged mild inflammation is always associated with HFD-induced obesity^26^ and, eventually, liver disease, as indicated by increasing serum liver enzyme levels and hepatic fat accumulation^18^. The overexpression of inflammatory markers, including NF-κB and iNOS, and the production of excessive NO were observed in obese mice^26^. Fruit vinegars, such as pineapple vinegar, have been identified as good sources of antioxidants and compounds with anti-inflammatory effects^6^. In this study, Nipa vinegar was able to suppress the expression of the inflammatory markers NF-κB and iNOS, leading to lower levels of NO in both the liver and fat pads. This result may be attributable to the phenolic acids present in Nipa vinegar. Furthermore, the up-regulation of AD by Nipa vinegar may also contribute to the anti-inflammatory effect, as AD inhibits inflammation by suppressing NF-kB activation^27^.

A recent study revealed the importance of microbial ecology in humans because of its effect on energy homeostasis^17^. Clinical evidence has correlated obesity with increased Firmicutes and decreased Bacteroidetes and Verrucomicrobia populations in the gut^17^. However, a decreased gut Firmicutes/Bacteroidetes ratio and increased Proteobacteria were recorded in women who lost weight after consuming fermented kimchi^5^. Firmicutes were determined to be able to ferment unabsorbed carbohydrates in the gut. Therefore, a high population of Firmicutes in obese subjects was linked to extra energy extraction from non-digestible carbohydrates, which subsequently increased the amount of energy intake^16^. Both SH and NH exhibited effects similar to those of fermented kimchi; that is, in NH-treated mice, the population of Firmicutes in the gut microbiota decreased, whereas those of Bacteroidetes, Proteobacteria and Verrucomicrobia increased. Additionally, the relative abundances of beneficial bacterial genera *Bacteroides, Lactobacillus, Parabacteroides* and *Akkermansia* increased, whereas those of *Blauti* and *Allobaculum* decreased when health returned to balance^5, 28^, as observed in SH- and NH-treated obese mice. The presence of mucin-degrading *Akkermansia* has been closely linked to weight loss and reduced inflammation^29^. More interestingly, *Clostridium*, a genus that includes a gut pathogen that promotes HFD-induced obesity^30^, was only detected in the gut microbiota profile of obese mice and was absent in SH- and NH-treated mice. Because the alterations in the gut microbiota were similar in both SH-and NH-treated mice, the organic acids, particularly acetic acid, may have been the major ingredients that contributed to these effect. Previous studies have reported that organic acids may regulate the gut microbiota by reducing digestive pH, increasing pancreatic secretion and exerting trophic effects on the gastrointestinal mucosa^31^.

Based on the above results, 2-ml/kg BW Nipa vinegar outperformed synthetic acetic acid vinegar in BW reduction, hyperlipidaemia and hepatoprotective effects, possibly because of the presence of metabolites, such as gallic acid and protocatechuic acid, which were previously detected in the Nipa vinegar used in this study^16^. The presence of these phenolic acids correlated with the anti-inflammatory effect of Nipa vinegar treatment that contributed to recovery from paracetamol-induced liver damage^16^. These phenolic acids may have also contributed to the recovery from obesity-linked oxidative stress, inflammation and damage in the livers of Nipa vinegar-treated obese mice. In contrast, only mild improvements in these parameters were observed in the obese mice treated with synthetic acetic acid vinegar, which contained only acetic acid. Gallic acid^32^ and protocatechuic acid^33^ have also been reported to possess anti-obesity and hyperlipidaemia effects on HFD-fed mice. These polyphenolic acids, which are present in Nipa vinegar at high concentrations, may help to further improve the serum lipid profile and reduce BW compared to synthetic acetic acid vinegar.

In summary, both synthetic acetic acid vinegar and Nipa vinegar reduced the food intake of obese mice and altered their gut microbiota, contributing to significant weight loss among the obese mice. Moreover, a high dose of Nipa vinegar achieved a significant reduction in BW, improved serum lipid profile, and reduced liver oxidation and inflammation in the obese mice relative to untreated mice. Thus, the active ingredients present in Nipa vinegar, particularly the polyphenols, further enhanced the anti-inflammatory, hypolipidaemic effect and weight loss in obese mice relative to those of synthetic acetic acid vinegar. Therefore, Nipa vinegar at high concentrations is a potential functional food ingredient with anti-obesity effects.

## Materials and Methods

### Preparation of Nipa vinegar

Fresh Nipa sap (collected from Pasar Borong Selangor, Malaysia) was filtered, Pasteurised and inoculated with *Saccharomyces cerevisiae 7013 INRA* (DSM, Netherlands) for 10 days. Then, the Nipa alcohol was inoculated with *Acetobacter acetiivar Europeans* (Culture collection centre of MARDI, Malaysia) for 40 days. The fermented Nipa vinegar was then filtered, titrated to 4% acetic acid and stored at 4 °C. Synthetic acetic acid vinegar was purchased from a local store (Tesco, Malaysia), titrated to 4% acetic acid and stored at 4 °C^10^.

### HFD-fed animals, Nipa treatment, and determination of BW and fat pad weight

This study was approved by the Institutional Animal Care and Use Committee (IACUC) of Universiti Putra Malaysia (UPM/IACUC/AUP-R097/2014). All animal procedures were performed according to a protocol approved by the IACUC of Universiti Putra Malaysia. C57BL/6 mice (7weeksold; n = 36), obtained from Monash University Malaysia Campus were separated into six groups and acclimatized for 7 days with a standard pellet diet (Mouse pellet 702-P from Gold Coin Co, Limited, Malaysia) and distilled water *ad libitum* (22 ± 1 °C; 12-h dark/light cycle). Then, the mice from groups 2 to 6 were given an HFD (D12451, 45% kcal fat, Research Diet, USA) for 33 weeks. The major components of both the standard pellet diet and HFD are listed in Table 2. From week 24 to the end of week 33 (10 weeks in total), mice from groups 2 to 6 were fed 200 µl of distilled water, 0.08-ml/kg BW synthetic acetic acid vinegar, 2-ml/kg BW synthetic acetic acid vinegar, 0.08-ml/kg BW Nipa vinegar or 2-ml/kg BW Nipa vinegar, respectively. The vinegar concentrations used in this study were selected based on the recommended amount (1 tablespoon of vinegar per day; low concentration) and that used in a previous study (high concentration)^6^. Mice fed a normal diet were maintained as a normal control. At week 33, all mice were euthanized. Blood samples were collected from their hearts by cardiac puncture and tested for glucose levels using a glucose metre. Serum was separated from whole blood and tested for serum cholesterol, TG, LDL, HDL and LDH using a biochemical analyser (Hitachi 902 Automatic Analyzer) and adapted reagents from Roche (Germany). Serum insulin and leptin levels were quantified using an enzyme-linked immunosorbent assay (ELISA) kit (R&D systems, USA). Liver and gonadal fat pads were harvested, weighed, immersed in formalin, fixed in paraffin, sectioned to 0.1 mm, stained with haematoxylin and eosin (H&E) and viewed under a *Nikon Eclipse 90i* microscope (Japan).

**Table 2:** Major components of the standard diet and HFD.

	Standard pellet (gm%) (Gold coin, Malaysia)	D12492 (gm%) (Research diet, USA)
Protein	22	26.2
Fiber	5	6
Carbohydrate	49	26.3
Fat	3	34.9
Others	21	6.6

### Quantitative real-time polymerase chain reaction (PCR) analysis

The differential expression of GLUT4, AD, and SREBP1 genes in the gonadal fat pads and GLUT2, NF-κB and iNOS in the livers of Nipa vinegar-treated mice or normal mice relative to those of untreated mice were quantified using quantitative real-time PCR (qRT-PCR) assay. Glyceraldehyde 3-phosphate dehydrogenase (GAPDH) and β-actin were used as housekeeping genes to normalize the expression levels of the above target genes. RNA from gonadal fat pads and liver was extracted using an RNeasy Mini Kit (Qiagen, Germany), and cDNA was synthesized using iScript™ Reverse Transcription Supermix (Bio-Rad, USA). qRT-PCR was performed using iTaq Universal SYBR Green Supermix (Bio-Rad, USA) on CFX-96 (Bio-Rad, USA). The primer sequences of the target and housekeeping genes are listed in supplementary Table 1. The PCR conditions for the standard curve and sample analyses were as follows: 1 cycle of 50 °C/2 min for uracil-DNA glycosylase (UDG) activation, 1 cycle of 95 °C/2 min for DNA polymerase activation, 40 cycles of 95 °C/2 s for denaturation, and 52 °C for 30 s for annealing and extension.

### Gut microbe composition profiling by Illumina 16S rRNA metagenomics sequencing

Faeces from untreated mice and those treated with synthetic acetic acid vinegar or Nipa vinegar (2 ml/kg BW) were collected at week 33, prior to the end of the experiment. DNA was extracted using a QIAamp DNA stool mini kit (Qiagen, Germany). The faecal microbiota populations were evaluated using Illumina 16S metagenomic sequencing with the 16S Amplicon PCR Forward Primer (5′-TCGTCGGCAGCGTCAGATGTGTATAAGAGACAGCCTACGGGNGGCWGCAG-3′) and 16S Amplicon PCR Reverse Primer (5′-GTCTCGTGGGCTCGGAGATGTGTATAAGAGACAGGACTACHVGGGTATCTAATCC-3′). The sequencing data were subjected to quality data pre-processing with quality value 20 (Q20) using a pair-end read merger. The processed data were subjected to taxonomic annotation via a homology search of the 16S microbial database (ftp://ftp.ncbi.nih.gov/blast/db/). The blast results were further analysed using MEGAN for taxonomic classification^34–37^.

### Statistical analysis

All experimental results are expressed as the mean ± standard deviation (SD). One-way analysis of variance (ANOVA) followed by Dunnett’s test (p < 0.05 was considered significant comparing to the untreated group) was performed using SPSS version 16 (USA) to compare the effects of low and high concentration of vinegar (synthetic acetic acid vinegar and Nipa vinegar) treated groups with untreated obesity group for all experiments.
